# Aligning Assessment With Intervention: An Inpatient Mental Health Initiative

**DOI:** 10.1111/inm.70374

**Published:** 2026-10-08

**Authors:** Makenzie Simon

**Affiliations:** ^1^ Michigan State University College of Nursing East Lansing Michigan USA

## Abstract

Aligning assessment and intervention decreases risk for violence against nurses working in psychiatric settings. There is an urgent need to ensure the well‐being of health care professionals who are on the frontline of mental health care. There is a notable gap in how nurses at the project site document and address these incidents. Despite existing tools, such as the Broset Violence Checklist (BVC), there is inconsistent application in real‐world scenarios. A robust approach that ensures risk assessment directly leads to timely and appropriate interventions is crucial. Reducing violence in mental health settings is vital for both staff safety and quality patient care. With proper implementation and staff education, the BVC Risk Bundle can play a key role in mitigating these risks. There was a statistically significant decrease in the number of injuries associated with BVC scores < 2, and an improvement in the timeliness of BVC score documentation after an injury with a *p* value of 0.04. While the target of 100% compliance was not met, the initiative led to an 11.1% improvement in the alignment of BVC scores with documented incidents, as well as a 0.7‐h reduction in documentation time. Despite these safety improvements, barriers remain. Aligning assessment with intervention is not merely a procedural change; it is a vital step toward supporting safer work environments for mental health professionals and the patients who are in their care.

The rates of physical violence against nurses worldwide support the need to improve healthcare's approach to violence prevention. In 2022, the State of Michigan reported an annual non‐fatal occupational injury rate of 7.7 per 100 full‐time employees in psychiatric and substance abuse hospitals (U.S. Bureau of Labor Statistics 2023). In 2 months, the rate of staff injury at the project site, including all inpatient mental health workers, was approximately 4.4 per 500 workers, which was below average for non‐fatal workplace violence. However, the importance of reduction remains (Thompson 2023). Nurses in psychiatric wards are more often exposed to physical attacks when compared to their nursing peers (Babiarczyk et al. 2020). However, in inpatient psychiatric nursing care, there is a gap in documentation regarding violence assessment and violence treatment (Brathovde 2020). Specific approaches to violence prevention, such as risk assessment implementation and education and training, are known elements for violence prevention (Karlsen et al. 2022). At the project site, the application of the Brøset Violence Checklist (BVC) Risk Bundle was anticipated to help support safe and accurate documentation that aligns with organizational policies and regulatory requirements (The Joint Commission 2021).

## 1 Background and Organization Gap Analysis

Nurses in psychiatric wards are more often exposed to physical attacks compared to their peers. A known gap in practice impacts the future treatment and prevention of physical violence in documentation related to violence assessment and intervention. At the project site, the application of the Broset Violence Checklist (BVC) Risk Bundle was anticipated to support safe and accurate documentation aligned with organizational policies and regulatory requirements.

To improve clarity, the relationship between the assessment tool (BVC), the inpatient units and organizational policy must be explicitly defined. The BVC serves as the standardized risk assessment tool used across all eight inpatient mental health units. Organizational policy requires that BVC scores guide clinical decision‐making regarding treatment and prevention of physical violence; however, inconsistencies in application across units contributed to a gap between documented risk assessment and actual events of physical violence. This disconnect highlights the need for a structured process linking assessment, interpretation, and intervention.

Data from the project site demonstrated misalignment between documented BVC scores and actual injury events, indicating a critical practice gap.

In December 2023, the project site's Workplace Safety Committee (in collaboration with the Quality Department and Enterprise Risk Management [ERM]) identified the need for a quality improvement (QI) initiative to re‐educate nursing staff on using the BVC. The chart audit findings led the hospital and residential services medical director, as well as other stakeholders, to express concern regarding staff injury reporting and the compliance issues that occurred as a result. At that time, organization wide education was implemented on the use, expectation and projected impact of appropriate use of the BVC tool would have on early detection and treatment of physical violence.

### 1.1 The Broset Violence Checklist as a Risk Assessment Tool

The BVC is a brief, evidence‐based assessment used to predict short‐term risk of patient violence within a 24‐h period. It evaluates six observable behaviours and assigns a cumulative score corresponding to risk level. While embedded in the electronic health record (EHR), its effectiveness depends on accurate scoring and appropriate clinical response. In this project, the BVC was conceptualized not only as a documentation requirement but as a decision‐support tool intended to trigger timely and appropriate interventions to prevent and manage physical violence.

The BVC assesses for the presence or absence of six risk factors, including (a) confusion, (b) irritability, (c) boisterousness, (d) verbal threats, (e) physical threats, and (f) attacking objects. The checklist uses these factors to predict the potential for violence within the subsequent 24 h. A score of 0 is low risk, scores of 1–2 indicate moderate risk and scores between 3 and 6 indicate high risk (Almvik et al. 2000; Ilarda et al. 2023). According to the project site's 2023 *Workplace Safety Committee Report*, of the 107 documented injuries, three incidents had no BVC score documented; 40 had a BVC score of 0; and 16 had a BVC score of 1. The gap in practice is noted; the patient who inflicted a staff injury had a recorded BVC score of < 2 in the EHR; this does not align with a defined risk of violence as supported by literature and organizational policy (Pérez et al. 2021; Thompson 2023). Nursing staff were erroneously reporting staff injuries inflicted by patients in the incident‐reporting software; the Electronic Health Record (EHR) BVC score does not align with the behaviour described.

Despite the availability of the BVC, several process gaps were identified. First, risk assessment did not consistently lead to intervention, limiting the tool's clinical impact. Second, discrepancies between BVC scores and incident reports suggested either delayed or inaccurate assessment. Third, staff's understanding of how BVC scores should inform care varied across units. These findings indicate that the issue was not solely the presence of a tool, but the absence of a standardized process connecting assessment to action.

### 1.2 Significance of the Practice Problem

The implementation of the BVC Risk Bundle across inpatient services was grounded in the need to standardize violence prevention practices. Variability across units created inconsistencies in how risk was assessed and addressed. A unified approach ensured that all staff operated under consistent expectations for assessment, documentation and intervention, supporting both patient safety and staff well‐being. In healthcare, violence most commonly occurs when the aggressor is a patient (Raveel and Schoenmakers 2019). Data collected from the project site between March 2023 and May 2023 concluded that in 107 reported incidents, patients were responsible for all the injuries inflicted on employees working in inpatient mental health units.

For staff caring for a mental health patient, how does the BVC Risk Bundle, compared to current practice, affect the documented risk for violence scores over 10 weeks? A reduction of physical violence in the mental health setting is crucial for the delivery of safe and quality nursing care (Escue et al. 2023). Patient violence can be predicted and prevented with a timely evidence‐based nursing assessment tool (Inuenwi et al. 2023). Addressing the threat of violence by assessing risk factors provides an opportunity to prevent staff injury (Lockertsen et al. 2021; Yuniati et al. 2020). According to National Nurses United (2024), only one in three nurses feel their employer has a clear way to report incidents. The BVC Risk Bundle provides a method by which to promote safety at work, facilitates an interdisciplinary approach and demonstrates organizational support for staff who are physically injured by patients (Renzi 2020).

### 1.3 Summary of the Evidence

Registered nurses (RNs) are generally responsible for completing the BVC Risk Assessment. Users report satisfaction with the convenience, performance and the average time for completion of 2 min (Kös et al. 2024; Dickens et al. 2020; Hvidhjelm et al. 2023; Inuenwi et al. 2023; Brathovde 2020; Yuniati et al. 2020). Evidence suggests nurses across the healthcare spectrum lack evidence, expertise and concern for early warning signals of impending violence and accompanying prevention approaches. Known approaches to violence prevention include (a) using a risk assessment tool and (b) initial and ongoing education on violence risk factors, prevention and management (Karlsen et al. 2022; Hou et al. 2022). As adult learners require multiphase education to positively change skills, knowledge and behaviours as health professionals (Inuenwi et al. 2023; Maguire et al. 2022), simply changing policies and educating staff is not enough to decrease workplace violence (Hendrickson 2022; Spencer et al. 2023).

The literature indicates that the most prominent type of workplace violence is Type II (customer‐on‐worker), which is perpetrated by patients, family members or visitors (Byon et al. 2021; Soliman et al. 2023). Interviewed nurses admit that after experiencing violence, they are afraid. Experiencing violence in the workplace can result in lower quality care (Babiarczyk et al. 2020; Odes et al. 2022). However, the literature is contradictory regarding the rate of physical violence reports in healthcare; some report that as much as 88% of nurses do not report violent altercations, while others claim that most physical injuries at work are documented for financial compensation (Buterakos et al. 2020; Kim et al. 2022). Barriers to reporting include lack of time to report; belief that violence is part of the job; an unstandardized reporting process; ambiguity on the reportability of violence, including an unclear definition of violence; and lack of management support (Byon et al. 2021; Huang et al. 2022).

For the BVC to be effective, assessment must systematically trigger intervention. Moderate‐risk scores should prompt increased observation or de‐escalation strategies, while high‐risk scores require immediate safety planning. Without this linkage, the BVC functions primarily as documentation rather than a preventative clinical tool.

### 1.4 Quality Improvement Framework

The Plan‐Do‐Study‐Act (PDSA) model is a QI process that allows an organization to test and analyse modifications to determine if a proposed change is improving a process (Eby 2021). This model was the best fit for the implemented QI project, as the project site needed to address a documentation gap with multiple solutions. Project leaders conducted a PDSA cycle prior to the project implementation, but there were continued gaps in documentation. The data was assessed from the first cycle to inform the project PDSA cycle. The assessment concluded that more than education was needed. The resulting recommendations were that staff be consistently reminded and encouraged to support the change in policy that was implemented in the first cycle (Hendrickson 2022; Spencer et al. 2023). The BVC Risk Bundle was anticipated to assist in the organization's immediate needs and align policy to practice. Prior cycles indicated that education alone was insufficient, reinforcing the need for stronger alignment between policy, practice and clinical behaviour. Further cycles will need to be conducted to address other causes for this gap.

### 1.5 Project Description

The project setting and population include eight inpatient mental health units. *Inpatient mental health units* are defined as facilities that provide around‐the‐clock care to adults or adolescents who are in acute mental health crises. Inclusion criteria included nursing and non‐nursing staff, as well as case managers, activity therapists, psychiatric technicians, RNs and clinical service managers (CSMs). The project site was a behavioural health institution located in Michigan that serves patients across the spectrum of behavioural health care. Before project implementation, the project leader assessed the LMS transcript for completion of historical BVC education and attestation of understanding.

On the first day of project implementation, the project leader conducted in‐person education on the BVC Risk Bundle expectations for each of the eight units. Education included the organization's policies and expectations regarding documentation. The policy supports that all documentation should be completed in real time, and all new behaviour that is assessed in the BVC should result in an updated BVC score. Intervention education included that moderate‐risk scores should prompt increased observation or de‐escalation strategies, while high‐risk scores require immediate safety planning. Injury education included that all physical injuries must be reported immediately and include all relevant antecedents. In‐person education was repeated five times. To reach the greatest number of employees, the project leader completed in‐person rounding on all three shifts at least once. The rounding was conducted from June 1, 2024, to August 8, 2024, between the hours of 7:00 a.m. and 3:30 p.m., 3:30 p.m. to 12:00 a.m. and 12:00 a.m. to 7:30 a.m. to (a) educate staff on the proper use of the BVC according to the project site policy; (b) to encourage reporting of workplace injuries within the incident report software; and (c) to provide the clinical literature when applicable. The project leader posted flyers with appropriate dates and expectations, as well as sending an Outlook email four times to all impacted staff approximately every 3 weeks.

## 2 Results

The evaluation of the project consisted of qualitative and quantitative data. Multiple tools were utilized for data collection during the pre‐intervention and intervention phases, including a report programmed within the EHR to identify the total BVC Risk Score for all patients admitted daily in all eight inpatient units. The project also used another tool that applied incident reporting software with a filter to assess only physical assault within employee injury reports, including incidents of kicking, punching, pushing, pulling, biting, scratching and shoving. One incident of perceived severe emotional damage was also included. The third tool for data collection was the LMS system automated weekly report, which was programmed to measure the number of delinquent employees who met the inclusion criteria in the assigned education module related to the BVC policy at the project site. The quantitative data points were utilized to inform the qualitative data. The quantitative data gave context and additional information when not all qualitative data (e.g., patient or employee identifiers) were included in the EHR report (or incident report) to identify the patient or the employee reporting the injury. The pre‐intervention period data were compared to the intervention period data to evaluate the project's outcome. This project did not grant access to any identifiable patient or employee data for any individuals who did not already have access to this data through their job roles. Any data shared beyond standard data access was de‐identified. A team member removed all identifiers from the dataset before sharing them with the project leader, including names and all geographical subdivisions (i.e., unit name and all date elements for date of birth, date of admission and date of discharge). Dates of workplace injury and incident reports were coded based on the month only. Other data was de‐identified, including electronic mailing addresses, medical record numbers, patient names, employee names and employee identification numbers. BVC scores were expected to be completed prior to or at the onset of behavioural escalation; however, in some cases, documentation occurred after an incident. This delay limits the predictive value of the tool and highlights the importance of timely assessment in preventing escalation. The timeline of data collection relative to reported incidents is important for interpretation. The project emphasized not only accurate completion of the BVC but also its integration into clinical decision‐making, ensuring that identified risks resulted in appropriate and timely interventions.

### 2.1 Design and Instrumentation

The BVC has a predictive validity that is reported to be consistently strong, with studies finding inter‐rater reliability of 0.84 for a single item. A kappa value ranging from 0.48 to 1.0 with 100% agreement when assessing the presence or absence of attacking objects (Dickens et al. 2020; Ghosh et al. 2019) is also observed. In prior studies, the BVC predicted short‐term events occurring between 24 and 72 h after admission at a significant level, as well as predictive validities for almost all events until discharge. The mean BVC total score for the sample at admission was 2.30 (SD = 1.80), with an acceptable internal consistency (Cronbach's alpha = 0.76; Dickens et al. 2020; Kös et al. 2024). The project site has copyright permission to utilize the tool within the EHR. As a result of this project, staff caring for mental health patients will report all incidents of patient‐inflicted workplace violence and document BVC scores of 2 or greater in the EHR with these documented behaviours (Lockertsen et al. 2021; Pérez et al. 2021).

Members of the QI team retrieved monthly aggregate data from the incident reporting software and procured the aggregate data from the EHR four times throughout the implementation phase. The project leader obtained learner records from the LMS record for completion of the associated module every week. The learner delinquency reports were then sent to the CSMs for review every 3 weeks to encourage compliance with project site policy and provide visibility for continued delinquent employees (see Appendix A for all study variables). A member of the QI team synthesized the staff injury data and the BVC score data to assess compliance and sent the de‐identified data to the project leader with all data from pre‐ to post‐implementation (see Appendix B for data source, access and storage information).

### 2.2 Data Analysis

The data analysis included descriptive and diagnostic analyses. The initial analysis totaled the number of reported/documented injuries and compared the injury rate in the pre‐intervention phase to the intervention phase. There were 293 unique employees working within the eight inpatient mental health units to which the data applied; in this group, there were 55 injuries in the 80‐day pre‐intervention phase. If this injury rate remained the same for 1 year, the injury rate would be 0.86 per 293 employees per year. There were 47 injuries in the 70‐day intervention phase (the same 293 unique employees applied). If this injury rate is maintained for 1 year, the injury rate would be 0.83 per 293 employees per year. Many factors impact the injury rate, including staffing ratios, patient acuity, time of year and availability of coping skills (Inuenwi et al. 2023; Spencer et al. 2023). The injury rate at the project site remained below the State of Michigan's average in the mental health and substance use hospital category (7.7 per 100 full‐time employees per year; U.S. Bureau of Labor Statistics 2023). There were a total of 55 injuries in the pre‐intervention phase and 47 injuries in the intervention phase. Missing patient information and required BVC data were handled with listwise deletion, resulting in the exclusion of 17 injury incidents in total between the pre‐intervention and intervention phases. Measured time elapsed between injury and BVC scores before injury (*t* [92] = 2.79, *p* = 0.006) and BVC scores at/after time of injury, exhibited significant positive skewness of variables. Measured time elapsed between injury and BVC scores was *t* (92) = 2.79, *p* = 0.006, and *t* (92) = 4.79 m, *p* = < 0.001; outliers were identified, as time elapsed was more than two standard deviations above or below the sample mean. Diagnostic analysis was completed when the data were concluding a result that the average number of hours between injury and the documented BVC score was greater than the outcome measure of 2 h. The diagnostic analysis compared the BVC score to the number of average hours needed to complete the injury report. It was noted that there was a statistically significant correlation between fewer hours needed to report an injury when the BVC score increased by 1 point.

#### 2.2.1 BVC Risk Total Score Entries

The project's primary outcome was that the proportion of patients who caused injury to staff had a BVC score ≥ 2 documented in the EHR at or after the time of the staff injury (target value: 100%). In the pre‐intervention phase, 46 patient‐initiated employee injuries were analysed. Incomplete records were removed from the dataset for statistical analysis. Incomplete was defined as no BVC score documented or standard deviations above or below the group means of time elapsed between BVC score documentation and the time of injury. Nineteen EHR records had BVC scores of < 2 at/after the documented employee injury. Twenty‐seven EHR records had a BVC of 2 or greater at/after the documented employee injury. During the intervention phase, 47 employee injuries were documented. Incomplete records were removed from the dataset for statistical analysis. Thirteen records had a BVC score of < 2 at/after injury. Thirty records had a BVC of 2 or greater at/after injury. There was an 11.1% decrease in the number of documented employee injuries with a BVC score in the EHR that was < 2. This is a statistically significant improvement (*X* ^2^ [1, *N* = 85] = 2.19, *p* = 0.04; see Figure 1). The outcome measure was not met. There are several potential reasons that < 100% of patients had a BVC score > 2, including unprovoked or sudden onset violent behaviour; inaccurate account of time by the employee who reported the injury; violence occurring at the time of admission; or the subjectivity of the data collected by the BVC.

**FIGURE 1:**
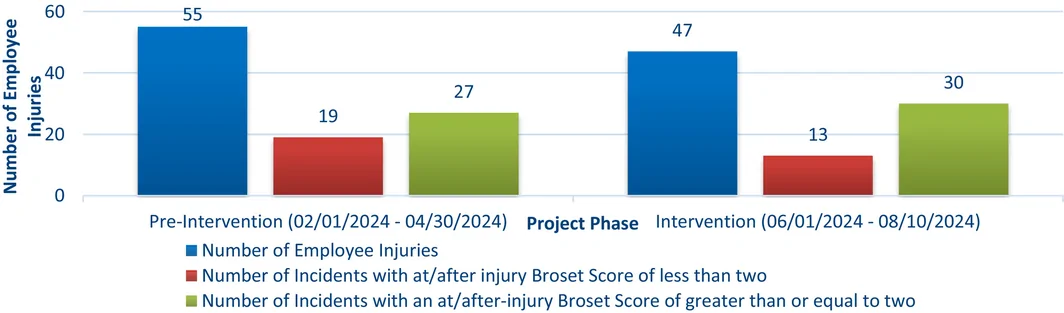
Employee injury incidents with aligned Brøset scores.

#### 2.2.2 Employee Injury Incident Report Data

The project outcome was that employees who report a patient‐inflicted injury were anticipated to demonstrate policy‐driven use of the BVC to score the patient's imminent risk for violence (target value: 100%). Published in 2023, the project site's policy supports documenting the BVC score in the EHR every 8 h and within 2 h of a change in patient condition. A *change in patient condition* for this project is defined as inflicting an injury upon an employee. During the pre‐intervention period, the mean elapsed time between the injury and the BVC at/after the injury was 5.4 h. During the intervention phase, the mean elapsed time between the injury and the BVC score at/after the injury was 4.7 h, with a mean difference in elapsed time between pre‐intervention and intervention phases of 0.7 h. The target outcome was not met with potential barriers. Possibilities include (a) other tasks with higher priority at the time of employee injury; (b) pressing priorities with other patient care needs; or (c) not documenting the precise time of the injury. Additional investigation was completed regarding the lack of timeliness in reporting. It was concluded that with less severe injuries resulting in a score < 2, there was more time between the score and report of injury, and the higher scores were documented closer to the reported injury time. This decrease in time was statistically significant *t* (85) = 2.19, *p* = 0.031, *d* = 242.64, with a correlation between elapsed time and BVC score (see Figure 2).

**FIGURE 2:**
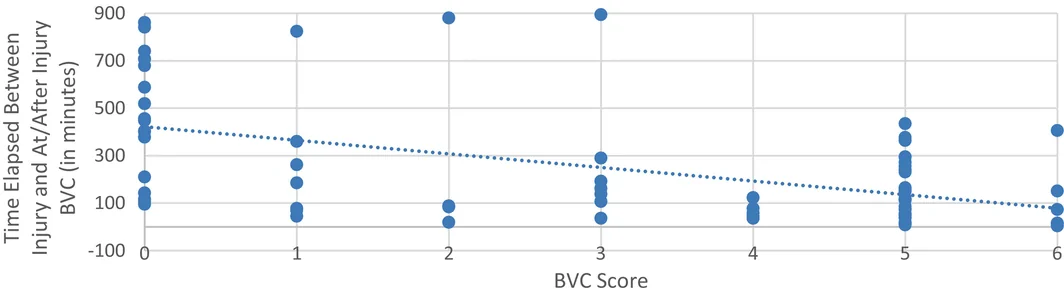
Time elapsed between BVC score and documented employee injury.

### 2.3 Employee Education Compliance

The LMS was assessed for staff who were delinquent in their assigned BVC education (target value: 0%). The percent of total delinquency among staff actively employed at one of the eight inpatient mental health units as a psychiatric technician or RN was described and compared to the pre‐intervention period of February 1, 2024, through April 30, 2024, as well as the intervention period of June 1, 2024, through August 11, 2024. Compliance within the LMS is not recorded until the next day, so an additional day was added to the intervention period. On May 31, 2024, 15.5% of all staff who met inclusion criteria were delinquent on the assigned education. By the end of the project (August 11, 2024), only 3.2% of employees remained delinquent in the assigned education. There was a reduction in delinquent employees in the assigned education by 12.3%. The total number of delinquent staff includes 16 unique staff on four units. All applicable CSMs are aware of staff that remain delinquent and confirm that these employees will continue to be prompted in the LMS to their assigned BVC education.

## 3 Implication for Nursing and Health Care

The practice change will be sustained at the project site through organizational policy and sustained information sharing by the Workplace Safety Committee and ERM. The organization continues to monitor employee injury reports and additional ways to support the nursing staff to accurately complete incident reports that include all necessary information, as well as promptly completing EHR documentation and showing confidence in the correct implementation of the violence prediction tool. As psychiatric patient acuity continues to rise nationally, it is important to have nursing staff buy‐in (McBain et al. 2022); increased compliance with documentation expectations may have a real impact on incidents of workplace violence. Recommendations for further practice include adding the BVC checklist in more places within the EHR to ease documentation burden. The integration of the BVC checklist within the restraint navigator and the Medication Administration Record could increase the convenience and, therefore, the compliance potential of updated BVC scores as the patient's condition changes. The Medication Administration Record is associated with emergency medication given to patients who show signs of danger to themselves or others. There are financial implications when considering integrating the flowsheet with the EHR that need careful consideration. The cost of adding the BVC flowsheet in more areas, as well as the cost of employee injuries, should be compared to assess if the potential for increased BVC documentation could decrease the rate of employee injuries. If this is the case, implementing these procedures could decrease the organization's costs related to employee injuries.

## 4 Conclusion

In summary, the evaluation results indicate that the project had successes outside of meeting the project outcomes. There was a significant decrease in the number of patients with BVC scores < 2 that inflicted injury on an employee. It was noted that there is an increased risk of violence by 3.4 times for each point scored on the BVC; quick interventions are imperative to prevent injury (Brathovde 2020; Dickens et al. 2020). The project results also noted a significant reduction in the elapsed time that the BVC score is documented in the EHR at/after a reported injury. Organizational policies support the project‐defined outcomes that all BVC scores should be updated upon a change to the patient condition. On average, there continued to be more than 4‐h lag between the reported injury and documentation of the BVC score. One potential barrier to meeting project outcomes is the limited time for the project and patient care priorities outside of documentation. Continued efforts by nursing staff to improve the timeliness of BVC score documentation would be beneficial for further progress in predicting and managing patient violence.

## 5 Relevance to Clinical Practice

This quality improvement project made a positive contribution to the current body of knowledge regarding the use of evidence‐based tools to document risk for injury and report violence in healthcare settings. The prediction and management of patient violence is crucial in providing a safe work and care environment for health care professionals and patients. Education for staff across the healthcare spectrum has been shown to empower staff and improve patient outcomes; this work contributes to those findings.

## Funding

The author has nothing to report.

## Conflicts of Interest

The author declares no conflicts of interest.

## Data Availability

Research data are not shared.

## Appendix B Ethical and Regulatory Issues

**Table:**

Variable	Data source	Data access personnel	Data storage and access
BVC risk total scores and date/time entries	Epic report	Team member (capture report) Team member (review, de‐identify and synthesize)	Exported reports were transferred electronically to a team member via Outlook with password protection. The team member merged Epic report data with Verge report data and de‐identified the resulting data collection into an Excel spreadsheet. The data collection spreadsheet was password‐protected and stored on a team member's OneDrive through the project site. The OneDrive was password‐protected with the Health Insurance Portability and Accountability Act and Health Information Technology for Economic and Clinical Health Act firewalls. Access to this password‐protected spreadsheet was provided to the project leader once it was de‐identified.
Employee injury incident report data	Verge	Team member (capture report) Team member (review, de‐identify and synthesize)	The exported report was transferred electronically to a team member via Outlook with password protection. The report was stored on the team member's OneDrive through the project site, protected with the Health Insurance Portability and Accountability Act and Health Information Technology for Economic and Clinical Health Act firewalls. Upon merging in Epic report data, recoding de‐identified fields and completing data collection calculations (e.g., time elapsed between BVC documentation and incident data), the team member de‐identified the resulting Excel spreadsheet and provided the project leader access to the de‐identified, password‐protected spreadsheet.
Employee education compliance	HealthStream	Project leader (capture report) Unit managers (review reports and communicate with staff as appropriate)	Exported reports with learner identity, including name, employee identification number and assigned unit, were electronically transferred to unit managers individually via Outlook; the report was then stored on the project leader's OneDrive through the project site, which was protected with the Health Insurance Portability and Accountability Act and Health Information Technology for Economic and Clinical Health Act firewalls; it was also password protected.

## References

[inm70374-bib-0001] Almvik, R. , K. Rasmussen , and P. Woods . 2000. Brøset Violence Checklist. Risk‐Assessment. https://www.risk‐assessment.no/about‐risk‐assessment/.

[inm70374-bib-0002] Babiarczyk, B. , A. Turbiarz , M. Tomagová , R. Zeleníková , E. Önler , and D. Sancho Cantus . 2020. “Reporting of Workplace Violence Towards Nurses in Five European Countries–A Cross‐Sectional Study.” International Journal of Occupational Medicine and Environmental Health 33, no. 3: 325–338. 10.13075/ijomeh.1896.01475. 32235948

[inm70374-bib-0003] Brathovde, A. 2020. “Improving the Standard of Care in the Management of Agitation in the Acute Psychiatric Setting.” Journal of the American Psychiatric Nurses Association 27, no. 3: 251–258. 10.1177/1078390320915988. 32281900

[inm70374-bib-0004] Buterakos, R. , M. M. Keiser , S. Littler , and C. Turkelson . 2020. “Report and Prevent: A Quality Improvement Project to Protect Nurses From Violence in the Emergency Department.” Journal of Emergency Nursing 46, no. 3: 338–344.e7. 10.1016/j.jen.2020.02.010. 32389206

[inm70374-bib-0005] Byon, H. D. , K. Sagherian , Y. Kim , J. Lipscomb , M. Crandall , and L. Steege . 2021. “Nurses' Experience With Type II Workplace Violence and Underreporting During the COVID‐19 Pandemic.” Workplace Health & Safety 70, no. 9: 412–420. 10.1177/21650799211031233. 34344236

[inm70374-bib-0006] Dickens, G. L. , L. E. O'Shea , and M. Christensen . 2020. “Structured Assessments for Imminent Aggression in Mental Health and Correctional Settings: Systematic Review and Meta‐Analysis.” International Journal of Nursing Studies 104: 103526. 10.1016/j.ijnurstu.2020.103526. 32062051

[inm70374-bib-0007] Eby, K. 2021. How to Implement PDSA in Your Organization. Smartsheet. https://www.smartsheet.com/content/plan‐do‐study‐act‐guide.

[inm70374-bib-0008] Escue, J. , F. Gutierrez , J. Batts , M. Lumagui , and V. Oloan . 2023. “Implementing Effective Interventions Against Workplace Violence.” Critical Care Nursing Quarterly 46, no. 3: 255–270. 10.1097/cnq.0000000000000457. 37226917

[inm70374-bib-0009] Ghosh, M. , D. Twigg , Y. Kutzer , A. Towell‐Barnard , G. De Jong , and M. Dodds . 2019. “The Validity and Utility of Violence Risk Assessment Tools to Predict Patient Violence in Acute Care Settings: An Integrative Literature Review.” International Journal of Mental Health Nursing 28, no. 6: 1248–1267. 10.1111/inm.12645. 31454144

[inm70374-bib-0010] Hendrickson, S. W. 2022. “Changing Attitudes About Workplace Violence: Improving Safety in an Acute Care Environment.” Journal of Healthcare Risk Management 42, no. 2: 39–43. 10.1002/jhrm.21524. 36125187

[inm70374-bib-0011] Hou, Y. , M. Corbally , and F. Timmins . 2022. “Violence Against Nurses by Patients and Visitors in the Emergency Department: A Concept Analysis.” Journal of Nursing Management 30, no. 6: 1688–1699. 10.1111/jonm.13721. PMC9795924 35700325

[inm70374-bib-0012] Huang, L. , H. Chang , X. Peng , F. Zhang , B. Mo , and Y. Liu . 2022. “Formally Reporting Incidents of Workplace Violence Among Nurses: A Scoping Review.” Journal of Nursing Management 30, no. 6: 1677–1687. 10.1111/jonm.13567. 35213934

[inm70374-bib-0013] Hvidhjelm, J. , L. L. Berring , R. Whittington , P. Woods , J. Bak , and R. Almvik . 2023. “Short‐Term Risk Assessment in the Long Term: A Scoping Review and Meta‐Analysis of the Brøset Violence Checklist.” Journal of Psychiatric and Mental Health Nursing 30, no. 4: 637–648. 10.1111/jpm.12905. 36718598

[inm70374-bib-0014] Ilarda, E. , P. McIlveen , A. Tynan , and A. Senz . 2023. “Emergency Department Staff Experiences of the Brøset Violence Checklist.” Journal of Advanced Nursing 80, no. 5: 2027–2037. 10.1111/jan.15961. 37975436

[inm70374-bib-0015] Inuenwi, B. , L. Lommel , S. B. Peter , and A. Carley . 2023. “Increasing Understanding and Perceived Confidence of Nurses Working in an Emergency Department in Assessing Patients at Risk of Violent Behavior.” Clinical Nurse Specialist 37, no. 3: 139–143. 10.1097/nur.0000000000000740. 37058705

[inm70374-bib-0016] Karlsen, I. L. , D. R. Andersen , S. Ø. Jaspers , L. P. Sønderbo Andersen , and B. Aust . 2022. “Workplace's Development of Activities and Action Plans to Prevent Violence From Clients in High‐Risk Sectors.” Journal of Interpersonal Violence 38, no. 1–2: 872–904. 10.1177/08862605221086633. 35465743

[inm70374-bib-0017] Kim, S. , M. R. Lynn , M. Baernholdt , R. Kitzmiller , and C. B. Jones . 2022. “How Does Workplace Violence–Reporting Culture Affect Workplace Violence, Nurse Burnout, and Patient Safety?” Journal of Nursing Care Quality 38, no. 1: 11–18. 10.1097/ncq.0000000000000641. 36409656

[inm70374-bib-0018] Kös, T. , P. Bräunig , and J. Hausam . 2024. “The Predictive Validity of the V‐Risk‐10 and BVC Among Involuntarily Admitted Patients.” Frontiers in Psychiatry 15: 1342445. 10.3389/fpsyt.2024.1342445. PMC10929738 38476613

[inm70374-bib-0019] Lockertsen, Ø. , S. Varvin , A. Færden , and S. K. Vatnar . 2021. “Short‐Term Risk Assessments in an Acute Psychiatric Inpatient Setting: A Re‐Examination of the Brøset Violence Checklist Using Repeated Measurements–Differentiating Violence Characteristics and Gender.” Archives of Psychiatric Nursing 35, no. 1: 17–26. 10.1016/j.apnu.2020.11.003. 33593511

[inm70374-bib-0020] Maguire, T. , B. McKenna , and M. Daffern . 2022. “Establishing Best Practice in Violence Risk Assessment and Violence Prevention Education for Nurses Working in Mental Health Units.” Nurse Education in Practice 61: 103335. 10.1016/j.nepr.2022.103335. 35390550

[inm70374-bib-0021] McBain, R. K. , J. H. Cantor , and N. K. Eberhart . 2022. “Estimating Psychiatric Bed Shortages in the US.” JAMA Psychiatry 79, no. 4: 279–280. 10.1001/jamapsychiatry.2021.4462. 35171208

[inm70374-bib-0022] National Nurses United . 2024. “Nnu Report Shows Increased Rates of Workplace Violence Experienced by Nurses.” https://www.nationalnursesunited.org/press/nnu‐report‐shows‐increased‐rates‐of‐workplace‐violence‐experienced‐by‐nurses#:~:text=NNU%E2%80%99s%20major%20findings%20on%20workplace,unit%20in%20the%20previous%20year.

[inm70374-bib-0023] Odes, R. , S. Chapman , S. Ackerman , R. Harrison , and O. Hong . 2022. “Differences in Hospitals' Workplace Violence Incident Reporting Practices: A Mixed Methods Study.” Policy, Politics & Nursing Practice 23, no. 2: 98–108. 10.1177/15271544221088248. PMC9234774 35317690

[inm70374-bib-0024] Pérez, R. , O. Torrecilla , S. García , et al. 2021. “Factors Associated With the Use of Mechanical Restraint in a Mental Health Hospitalization Unit: 8‐Year Retrospective Analysis.” Journal of Psychiatric and Mental Health Nursing 28, no. 6: 10521064. 10.1111/jpm.12749. 33657672

[inm70374-bib-0025] Raveel, A. , and B. Schoenmakers . 2019. “Interventions to Prevent Aggression Against Doctors: A Systematic Review.” British Medical Journal Open 9, no. 9: e028465. 10.1136/bmjopen-2018-028465. PMC6756459 31530592

[inm70374-bib-0026] Renzi, T. M. 2020. “The Effect of Leadership Styles on Project Implementation.” Open Journal of Leadership 9: 198–213. 10.4236/ojl.2020.94012.

[inm70374-bib-0201] Soliman, L. , A. Jain , J. Rozel , and J. Rachal . 2023. “Safe Spaces: Mitigating Potential Aggression in Acute Care Psychiatry.” FOCUS 21, no. 1: 46–51. 10.1176/appi.focus.20220069. PMC10172535 37205036

[inm70374-bib-0027] Spencer, C. , J. Sitarz , J. Fouse , and K. DeSanto . 2023. “Nurses' Rationale for Underreporting of Patient and Visitor Perpetrated Workplace Violence: A Systematic Review.” BioMed Central Nursing 22, no. 1: 134. 10.1186/s12912-023-01226-8. PMC10122798 37088834

[inm70374-bib-0028] The Joint Commission . 2021. “R3 Report Issue 30: Workplace Violence Prevention Standards.” https://www.jointcommission.org/standards/r3‐report/r3‐report‐issue‐30‐workplace‐violence‐prevention‐standards/.

[inm70374-bib-0029] Thompson, M. 2023. Brøset Score and Staff Injury. Project Site [Unpublished report].

[inm70374-bib-0030] U.S. Bureau of Labor Statistics . 2023. Incidence Rates of Nonfatal Occupational Injuries and Illnesses by Industry and Case Types, 2022. U.S. https://www.bls.gov/web/osh/table‐1‐industry‐rates‐national.htm.

[inm70374-bib-0031] Yuniati, W. , K. Rusca Putra , and D. Widasmara . 2020. “Factors Affecting the Success of Psychiatric Nurses in Conducting Risk Assessment of Violence Behavior.” International Journal of Nursing Education 12, no. 4: 252–256. 10.37506/ijone.v12i4.11259.

